# Impact of Work and Recreational Physical Activity on Prediabetes Condition among U.S. Adults: NHANES 2015–2016

**DOI:** 10.3390/ijerph18041378

**Published:** 2021-02-03

**Authors:** Lenin Pazmino, Wilmer Esparza, Arian Ramón Aladro-Gonzalvo, Edgar León

**Affiliations:** 1 School of Physical Therapy, Universidad de Las Américas, Quito 170124, Ecuador; lenin.pazmino@udla.edu.ec; 2 Facultad de Ciencias de la Educación, Pontificia Universidad Católica del Ecuador, Quito 170143, Ecuador; araladro@puce.edu.ec; 3 School of Medicine, Universidad de Las Américas, Quito 170124, Ecuador; edgar.leon.segovia@udla.edu.ec

**Keywords:** NHANES, prediabetes, physical activity, sedentary behavior

## Abstract

More minutes of physical activity (PA) accumulated during a day are associated with a lower risk of diabetes mellitus type 2. However, it is less known if distinct dimensions of PA can produce a different protective effect in the prevention of prediabetes. The aim of this study was to analyze the impact of work and recreational PA on prediabetes among U.S. adults during the period 2015–2016 using the National Health and Nutrition Examination Survey (NHANES) database. Individuals (*n* = 4481) with hemoglobin A1c (HbA1c) test values of 5.7% to 6.4% were included. A logistic regression multivariate-adjusted analysis was conducted to estimate the association between the odds ratios (ORs) and 95% confidence intervals (CIs) of prediabetes, with work and recreational PA. The prevalence of prediabetes among U.S. adults was lower in physically active individuals both at work (~24%) and recreational (~21%) physical activities compared to individuals who were not physically active (27 to 30%). Individuals lacking practice of recreational PA had a high risk of prediabetes (OR = 1.26, 95% CI: 1.080 to 1.466). PA may be a protective factor for prediabetes conditions depending on gender, age, ethnic group, waist circumference, and thyroid disease.

## 1. Introduction

Having blood glucose levels above normal, but below diabetes threshold is considered as prediabetes [1]. The American Diabetes Association (ADA) has established a range of plasma glucose as an indicator of this condition (100–125 mg/dL) [2]. Likewise, an additional diagnostic criterion of prediabetes includes glycosylated hemoglobin values (HbA1c) (5.7–6.4%) [2]. Whilst prediabetes is not considered a clinical illness, it can lead to a higher incidence of diabetes and cardiovascular disease [3,4].

The prevalence of prediabetes is growing worldwide, regardless of socioeconomic status. The values vary depending on geographic region and study population. For instance, 12.19% of prediabetics was reported in the Eastern Mediterranean Region [5]. In France the prevalence was 28.6% using the ADA criteria [6]. In the U.S., a study on Mexican Americans reported a prevalence of 32% [7]. Meanwhile, in Peru, this value was 53.6% [8]. Among adolescents with undiagnosed prediabetes, it can reach up to 14.3% [9], and, in overweight and obese school children, the incidence of prediabetes was 62.7% [10]. 

Some authors have identified identical risk factors amongst diabetes and prediabetes [11]. Notwithstanding, it has been established that patients with prediabetes present metabolic abnormalities, such as dysglycemia, dyslipidemia, hypertension, obesity, insulin resistance, procoagulant status, endothelial dysfunction, oxidative stress, and inflammation [12]. The changes in diet and practice of physical activity (PA) are the most relevant protective factors involved in lifestyle modification [13].

Work PA includes paid or unpaid work, studying or training, household chores, and yard work. Recreational PA involves the practice of sports, fitness, and relaxation activities (Yoga, Tai Chi, Feldenkrais). On one hand, work PA involves repetitive working postures, including lifting tasks within a low control environment and speed, that often are not given adequate recovery time. Whereas recreational PA is linked with self-controlled actions performed in short bouts with adequate rests [14]. Work-related PA has predicted health benefits, including lower obesity prevalence, lower rates of metabolic syndrome, and higher self-evaluated health [15,16]. PA has been shown that it is an effective, safe, and low-cost approach to reduce the risk of developing type 2 diabetes. Furthermore, the benefits achieved after one year of intervention can be maintained up to three years in the follow up phase. This can lead to modest weight reduction, as well as favorable cardiovascular risk factors changes [17]. However, most studies refer to well established exercise programs for a certain time-period [17,18]. Thereby, the effect of recreational and work PA on prediabetes has been relatively unexplored. The objective of this study was to analyze if these types of PA constitute a protective factor for prediabetes. For instance, a recent study showed that recreational PA was associated with lower prevalence of obesity, diabetes/prediabetes, and hyperlipidemia [19]. Thus, we hypothesize that recreational PA constitutes a protective factor more important than work PA for prediabetes. To achieve this objective, data from the 2015–2016 National Health and Nutrition Examination Survey (NHANES) database was analyzed.

## 2. Materials and Methods

### 2.1. Data Collection and Study Population

The NHANES is a program of studies conducted to understand the health and nutritional status of the United States population since the 1960s. The survey includes interviews and physical examinations in a nationwide representative sample of about 5000 participants each year. The NHANES gathers demographic, socioeconomic, dietary, and health-related information. The physical examination section involves medical, dental, and physiological measurements, as well as laboratory tests directed by qualified medical professionals [20].

The current study used data collected by NHANES during the period 2015–2016 with a 94.3% response rate of the interviewed sample [21]. A total of 15,327 individuals participated in the NHANES during 2015–2016. Participants that presented A1c test values were included in this study. We excluded participants with diabetes (HbA1c level > 6.4%), underage individuals, and participants without data about A1c, body mass index (BMI), race, educational level, cholesterol, measurements of blood pressure (BP), waist circumference, and thyroid disease. Finally, 4481 subjects older than 18 years were included in our analysis.

### 2.2. Assessment of Variables

#### Physical Activity (Exposure)

The Global Physical Activity Questionnaire (GPAQ) was used to measure PA in each NHANES participant. The concurrent validity of GPAQ has been evaluated in a recent systematic review showing poor to fair results. However, this questionnaire is widely used in the literature regarding the measurement of PA [22]. The PA questionnaire gathered information on work and recreational activities. This process was conducted through a Computer-Assisted Personal Interviewing (CAPI) system contacting the participant at home. The GPAQ categorized the intensity of the PA into vigorous activities that require hard physical effort and cause large increases in breathing or heart rate, and moderate-intensity activities which require moderate physical effort and cause small increases in breathing or heart rate [23]. The number of days and the minutes of PA completed were also asked in GPAQ for work/recreational and moderate/vigorous physical activity. We then combined the data obtained on vigorous and moderate PA on a single variable, considering whether the participant fulfilled the recommendations of the American College of Sports Medicine for each PA: minimum 20 min of vigorous PA a day, at least three times a week; minimum 30 min of moderate PA a day, at least five times a week; or minimum 30 min of walking a day, at least five times a week [24]. If the participant satisfied the PA recommendation, then it was coded as “Yes”, or if he did not as “No”, respectively, for work and recreational PA. 

### 2.3. Prediabetes (Outcome)

Prediabetes assessment was based according to A1c screening criterion on the diagnosis of prediabetes among U.S. adults of the American Diabetes Association (ADA). This criterion classifies prediabetes with an HbA1c level of 5.7–6.4%. In 2015, ADA advised that the A1c test should use a method that was certified by the National Glycohemoglobin Standardization Program (NGSP) and standardized or traceable to the Diabetes Control and Complications Trail (DCCT) reference assay [2]. After an evaluation of the NHANES laboratory and participant HbA1c data, the NGSP group concluded that the NHANES laboratories met the NGSP criteria for bias and precision [25].

### 2.4. Covariates

The interview collected demographic information on age, gender, race (Mexican, Hispanic, Non-Hispanic White, Non-Hispanic Black, and multiracial) and educational level (classified as school, higher school, technical education, and university or above). It included other covariates, such as total cholesterol levels (normal: <200 mg/dL, borderline: between 200–239 mg/dL, and high: 240 mg/dL or above), hypertension (identified as hypertensive if the mean systolic BP was ≥140 mmHg or diastolic BP was ≥90 mmHg or receiving antihypertensive medicine), BMI (low: <18.5 kg/m^2^, normal: 18.5–24.9 kg/m^2^, overweight: 25.0–29.9 kg/m^2^ and obese: >30.0 kg/m^2^), waist circumference measure (normal men: <94 cm, normal women: <80 cm, men at risk: >94 cm, women at risk: >80 cm), and thyroid disease (“Has a doctor or other health professional ever told you had a thyroid problem?”) [26].

### 2.5. Statistical Analysis

Descriptive statistics were used to explain the prevalence of comorbidities, while significant differences in work and recreational PA between groups were evaluated using bivariate chi squared tests. Multivariate-adjusted logistic regression analysis was conducted to estimate the association between PA (exposure) and prediabetes (outcome). The analysis carried in this model was adjusted for all covariates included in the study. Results from the logistic regression analysis are presented as odds ratios (ORs) and 95% confidence intervals (CI). A forest plot is also presented to illustrate the multivariate adjusted model significant odd ratios for work/recreational PA and prediabetes. The amount of multicollinearity in the set of Multivariate-adjusted logistic regression analysis was verified using Variance inflation factor (VIF). Low-moderate co-linearity was assumed when VIF <10. The level of statistical significance was set at *p* < 0.05. The statistical package (STATA version 15.0, StataCorp, LLC., College Station, TX, USA).

## 3. Results

### 3.1. Characteristics of Work and Recreational Physical Activity

Amongst the 4481 participants, 1945 (43.3%) performed work PA (Table 1), while 2275 (50.8%) performed recreational PA (Table 2) according to the data. The subjects who performed work PA, in comparison to those who did not, were males, between 18–64 years old, white ethnic background, completed at least technical education, without hypertension nor thyroid disease, were less overweight and obese, and men with waist circumference at risk. There was no association between cholesterol level and work PA (Table 1). Similarly, subjects who performed recreational PA in comparison to those who did not, were males, between 18–64 years old, white ethnic background, completed university, without hypertension nor thyroid disease, normal BMI, and women with normal waist circumference (Table 2).

### 3.2. Association between Prediabetes and Work/Recreational Physical Activity

The prevalence of prediabetes among U.S. adults was lower in physically active individuals both at work (23.9%) and recreationally (21.0%) compared to individuals who were not physically active (27 to 30%) (Table 3). The prediabetic individuals, in comparison to the non-prediabetic, were older than 65 years, black ethnic background, completed at least school, with hypertension, obese, men with waist circumference at risk, and subjects without thyroid disease. However, there was no association in terms of gender (Table 3).

**Figure 1:**
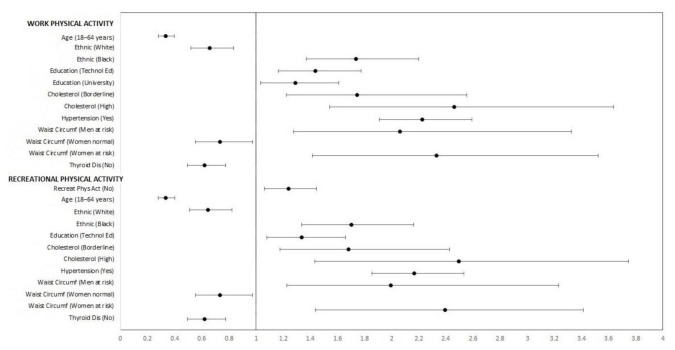
Forest plot of the multivariate adjusted model of significant odd ratios for work/recreational physical activity and prediabetes, in U.S. adults from NHANES 2015–2016.

Logistic regression multivariate-adjusted models showed a significant association between risk of prediabetes and PA status (x^2^ = 0.129 *p* = 0.003). In particular, we found a higher risk of prediabetes in individuals who lacked practice of recreational PA (OR = 1.26, 95% CI: 1.080 to 1.466). There was no significant association between prediabetes and work PA (OR = 1.02, 95% CI: 0.872 to 1.182). Furthermore, we found a lower risk of prediabetes in work and recreational PA in individuals between 18 and 64 years (OR = 0.33, 95% CI: 0.279 to 0.398), white ethnic (OR = 0.66, 95% CI: 0.519 to 0.836), women with normal waist circumference (OR = 0.73, 95% CI: 0.553 to 0.976), and individuals without thyroid disease (OR = 0.62, 95% CI: 0.495 to 0.776) (Figure 1). In contrast, black ethnic (OR = 1.74, 95% CI: 1.368 to 2.203), the entire education level (OR = 1.29–1.44, 95% CI: 1.029 to 1.774), borderline and high cholesterol (OR = 1.74–2.47, 95% CI: 1.217 to 3.655), hypertension (OR = 2.23, 95% CI: 1.909 to 2.601), and waist circumference at risk both for men (OR = 2.06, 95% CI: 1.276 to 3.338) and women (OR = 2.34, 95% CI: 1.415 to 3.538) showed high risk for prediabetes. (Figure 1). No independent variable was removed from the model because the assumption of non-colinality was met.

## 4. Discussion

The main finding of this study was that more work/recreational physically active individuals between 18 and 64 years old, white ethnic, women with normal waist circumference, and without thyroid disease, show a lower risk of prediabetes. In contrast, black ethnic, all education levels, borderline and high cholesterol, hypertension, and at-risk waist circumference in both men and women, were associated with high risk of prediabetes. To our knowledge, this is the first detailed analysis of the distinct dimensions of PA associated with prediabetic condition among U.S. adults demonstrating their protective effect.

We analyzed the association between demographic variables, risk factors of prediabetes (covariates) with work-related and recreational PA. There were significant associations between work-related and recreational PA with all covariates (Table 1 and Table 2), except cholesterol which was not associated with PA at work (Table 1).

Several studies have linked physical activity in lifestyle interventions with the presence of cardiovascular/metabolic risk factors, indicating its influence on the control of abdominal circumference, fasting glucose, triglycerides, and high-density lipoprotein (HDL) cholesterol, as well as in other pathophysiological mechanisms, such as a reduction in circulating levels of leptin, interleukin 18, and monocyte chemoattractant protein-1 (MCP-1) [27]. All these events contribute to a better control of insulin metabolism, endothelial activity, and pro-inflammatory factors.

PA, defined as “any activity that causes skeletal muscle contraction capable of increasing energy expenditure over resting levels and involves daily activities such as home, recreational, work, etc.” [28], has been shown to decrease the risk of high blood pressure. In fact, several studies indicate that moderate to high intensity aerobic activities reduce blood pressure by up to 10 mmHg, both in non-hypertensive and hypertensive people [29]. Among the physiological reasons for the action of PA on arterial hypertension are the effect of exercise on oxidative stress mechanisms and endothelial function. PA decreases both mechanisms, as well as increasing nitric oxide levels, improving the endothelial response [30]. These metabolic responses are also involved in the pathophysiology of other diseases, such as diabetes and prediabetes, leading to chronic inflammation and consequent endothelial damage can cause insulin resistance and impaired insulin secretion [31]. It is also known that PA can influence the secretion of insulin and glucagon, which in turn acts on the vascular endothelium and intermediate metabolisms [32]. Furthermore, it has recently been confirmed that PA reduces peripheral resistance to insulin, especially in prediabetic trained muscles [12].

A significant association was found between gender and work/recreational PA where fewer women performed work PA and fewer men performed recreational PA (Table 1 and Table 2). The relationship between PA and gender is multifactorial and includes body image self-perception as one of the most important points for a person to perform PA [33]. 

No association was found between cholesterol profile and PA at work, despite the fact that several studies show that moderate and high-intensity exercise (70% and 90% VO_2peak_) may be a strategy for improvement of lipoprotein metabolism in healthy subjects [34,35]. The relationship between the change in lipid profile and PA can be multifactorial, including diet. The results of the Lira and colleague indicate that, although high-intensity exercise generated low energy expenditure, it induced a reduction in LDL cholesterol and total cholesterol levels. However, these alterations in lipoprotein profiles induced by high-intensity exercise disappear when dietary carbohydrate intake is increased [36].

Prediabetic condition was associated with several demographic variables and all risk factors of prediabetes. Regarding demographic variables, 46% of subjects over 65 years old had prediabetes compared with 20% of subjects in the 18–64 years range who did not have this condition (Table 3). Several studies have demonstrated that the risk of getting prediabetes increases with age due to a decrease in insulin secretion with advancing age [37,38]. The overall ethnic prevalence of prediabetes was 26.4% (21.8–34.4%), with the highest estimate observed in Blacks (34.4%), followed by Mexican (27.8%) (Table 3). Current data from Patient Outcomes Research To Advance Learning (PORTAL), multisite cohort study of adults in the U.S., show that the overall age-standardized prevalence of prediabetes was 33.4%, with the highest estimate observed in Asians (37.1%), while Blacks ranked fourth within seven race/ethnicity categories with a prediabetes prevalence of 32.0% [39]. Despite the fact that, in both studies, the prevalence of prediabetes is similar among Blacks, it is shown that the prevalence by race/ethnicity categories is liable to vary when factors, such as the age of the subjects, are controlled. No association was found between gender and prediabetes condition (Table 3), however Mahat et al. [40] reported that there may be gender differences in the manifestations of prediabetes, which could be explained because the smaller muscle mass or physical fitness in women would lower insulin-stimulated glucose disposal and account for a higher rate of impaired glucose tolerance (IGT).

Prediabetic condition also was associated with cholesterol, blood pressure, BMI, waist circumference, and thyroid disease. In particular, the prediabetic prevalence was highest in subjects with high cholesterol (30.7%), hypertension (42.3%), and obesity (33.1%) (Table 3). The association between BMI and prediabetes was more pronounced in PORTAL study, with a prevalence >35% for any level of obesity [39]. The excess body fat may result in increased fat degradation, which results in the production of large amounts of free fatty acids (FFAs). In obese people, higher levels of FFAs in blood reduce the ability of liver tissue to process insulin-mediated glucose uptake and utilization, resulting in high blood glucose levels [41]. Hence, increased consumption of high-calorie food and decreased energy expenditure result in obesity and in a highest prediabetes risk. In Asians (people with a high prevalence of prediabetes), larger intra-abdominal fat and thicker truncal skin folds were consistent predictors of glucose intolerance and insulin resistance, with large waist circumference increasing the likelihood of prediabetes [42,43].

Augmented low-density lipoprotein (LDL) concentration can be detected in insulin resistant prediabetic people several years before the clinical diagnosis of type 2 diabetes [44,45]. Alterations of insulin sensitive pathways, increased concentrations of FFAs and low-grade inflammation all play a role and result in an overproduction and decreased catabolism of triglyceride rich lipoproteins of intestinal and hepatic origin. The observed changes in HDL and LDL are mostly due to this [45]. The underlying pathophysiology of diabetic dyslipidemia is complex, and, for a more extensive understanding, it is suggested to review [45].

Hypertension has been a strong predictor of prediabetes, with a high prevalence both for hypertension and prehypertension in prediabetes showing 45.3 to 51.2%, respectively [46,47]. One mechanism that explains the association between hypertension and prediabetes involves an enhanced angiotensin II activity which in turn triggers the renin-angiotensin-aldosterone system and affects the function of the pancreatic islets, resulting in islet fibrosis and decreased insulin synthesis and eventually leading to insulin resistance [48]. Another possible mechanism is that the impairment in the uptake of glucose due to delivery alteration of insulin and glucose to skeletal muscle by insulin resistance increased in patients with hypertension [49].

Regarding thyroid hormone alterations and glycemic problems for diabetes mellitus type 1, it is suspected that both may be related due to their similar autoimmune origin. The relationship between hypothyroidism and glucose disturbances has been described but requires further investigation [50]. Interestingly, we found that prediabetic individuals in comparison to non-prediabetics, were older than 65 years and without thyroid disease.

In this study, the prevalence of prediabetes among U.S. adults was lower in physically active individuals both at work (~24%) and recreational (~21%) activities compared to individuals who were not physically active (27–30%) (Table 3). According to grouped data, in England, in the year 2011, the prevalence of prediabetes was 35.5% based on HbA1c levels among the adult population [51]. Based on fasting glucose or HbA1c levels found in previous NHANES, 35% of U.S. adults over 20 years of age and 50% of those over 65 had prediabetes in 2005–2008 [52]. In addition, using the ADA definition (HbA1c levels), the prevalence was as high as 38% in 2012 [53]. Therefore, the results of this study from the NHANES 2015–2016 indicate the prevalence of prediabetes has decreased slightly, in addition to the fact that the data adjusted for levels of PA highlight that the prevalence of prediabetes is reduced when the population is active in the work and recreational environment (Table 3).

We found that individuals lacking practice of adjusted recreational PA, black ethnic, all education levels, borderline and high cholesterol, hypertension, and at risk waist circumference in both men and women, had a high risk of prediabetes (Figure 1). These results highlight the importance of personalized management of prediabetes risk factors and of the promotion of active lifestyle in U.S. adults.

The present study has several limitations. First, based on the cross-sectional study design, the causal relationship between PA and prediabetes is difficult to determine due to the survey data not coming from a PA deliberate practice intervention. Furthermore, responses from survey data to assess self-reported diet behavior can be an inaccurate information source. Second, the current study did not discriminate between work and recreational PA intensity (moderate and vigorous), with which it is difficult to know the intensity in which a lower risk of prediabetes can be achieved. Third, although a recent literature review of previous studies on the reliability and concurrent validity of the GPAQ, demonstrated good-to-very good test–retest reliability with time intervals that ranged from three days to two weeks, there are incomparable data to test the concurrent validity of the GPAQ using the data measured by accelerometers, pedometers, and PA log [22]. Therefore, until more evidence of the concurrent validity of the GPAQ is reached, the data from this questionnaire should be assumed with caution. Finally, the data used in this study were collected in 2015; therefore, the context and relationship between analyzed parameters should also be interpreted with caution. Despite these limitations, the strength of this study is that the objective and robust data used, provides a deeper understanding of the protective effect of the different dimensions of PA in the prevention of prediabetic condition.

## 5. Conclusions

In this nationwide and cross-sectional survey study, the prevalence of prediabetes among U.S. adults was lower in physically active individuals both at work and recreational activities compared to individuals who were not physically active. Prediabetes was associated with several demographic variables and risk factors of prediabetes. PA may be a protective factor for prediabetes conditions depending on gender, age, ethnic group, waist circumference, thyroid disease, and types of PA.

## Figures and Tables

**Table 1:** Characteristics of work physical activity in U.S. adults from the NHANES 2015–2016.

Variables		Work Physical Activity		*p*
		YES	NO	
		*n* (%)	*n* (%)	
		*n* = 1945	*n* = 2536	
		43.41%	56.59%	
	2015–2016			
Gender				0.001
	Males	1072 (50.42)	1054 (49.58)	
	Females	873 (37.07)	1482 (62.93)	
Age				0.001
	18–64 Years	1606 (45.41)	1931 (54.59)	
	>65 Years	339 (35.91)	605 (64.09)	
Ethnic Group				0.001
	Mexican	313 (42.53)	423 (57.47)	
	Hispanic	224 (37.71)	370 (62.29)	
	White	827 (53.60)	716 (46.40)	
	Black	373 (41.08)	535 (58.92)	
	Multiracial	208 (29.71)	492 (70.29)	
Education level				0.001
	Elementary School	356 (36.36)	623 (63.64)	
	High School	458 (47.12)	514 (52.88)	
	Technical Education	696 (51.33)	660 (48.67)	
	University	435 (37.05)	739 (62.95)	
Cholesterol				0.410
	Normal	1206 (44.10)	1529 (55.90)	
	Borderline	515 (42.85)	687 (57.15)	
	High	224 (41.18)	320 (58.82)	
Hypertension				0.011
	Yes	586 (40.67)	855 (59.33)	
	No	1359 (44.70)	1681 (55.30)	
BMI				0.003
	Low	24 (32.00)	51 (68.00)	
	Normal	508 (41.47)	717 (58.53)	
	Overweight	619 (41.97)	856 (58.03)	
	Obese	794 (46.54)	912 (53.46)	
Waist Circumference				0.001
	Men Normal	336 (46.86)	381 (53.14)	
	Men at Risk	736 (52.24)	673 (47.76)	
	Women Normal	107 (35.67)	193 (64.33)	
	Women at Risk	766 (37.27)	1289 (62.73)	
Thyroid Disease				0.017
	Yes	179 (38.25)	289 (61.75)	
	No	1766 (44.01)	2247 (55.99)	

* BMI, Body mass index: Low <18.5; Normal 18.5–24.9; Overweight 25–29.9, Obese ≥30. NHANES, National Health and Nutrition Examination Survey.

**Table 2:** Characteristics of recreational physical activity in U.S. adults from the NHANES 2015–2016.

Variables		Recreational Physical Activity		*p*
		YES	NO	
		*n* (%)	*n* (%)	
		*n* = 2275	*n* = 2206	
		50.77%	49.33%	
	2015–2016			
Gender				0.011
	Males	1122 (52.78)	1004 (47.22)	
	Females	1153 (48.96)	1202 (51.04)	
Age				0.001
	18–64 Years	1902 (53.77)	1635 (46.23)	
	>65 Years	373 (39.51)	571 (60.49)	
Ethnic Group				0.001
	Mexican	314 (42.66)	422 (57.34)	
	Hispanic	286 (48.15)	308 (51.85)	
	White	836 (54.18)	707 (45.82)	
	Black	463 (50.99)	445 (49.01)	
	Multiracial	376 (53.71)	324 (46.29)	
Education Level				0.001
	Elementary School	303 (30.95)	676 (69.05)	
	High School	426 (43.83)	546 (56.17)	
	Technical Education	729 (53.76)	627 (46.24)	
	University	817 (69.59)	357 (30.41)	
Cholesterol				0.044
	Normal	1428 (52.21)	1307 (47.79)	
	Borderline	577 (48.00)	625 (52.00)	
	High	270 (49.63)	274 (50.37)	
Hypertension				0.001
	Yes	606 (42.05)	835 (57.95)	
	No	1669 (54.90)	1371 (45.10)	
BMI				0.001
	Low	41 (54.67)	34 (45.33)	
	Normal	677 (55.27)	548 (44.73)	
	Overweight	762 (51.66)	713 (48.34)	
	Obese	795 (46.60)	911 (53.40)	
Waist Circumference				0.001
	Men Normal	426 (59.41)	291 (40.59)	
	Men at Risk	696 (49.40)	713 (50.60)	
	Women Normal	183 (61.00)	117 (39.00)	
	Women at Risk	970 (47.20)	1085 (52.80)	
Thyroid Disease				0.001
	Yes	202 (43.16)	266 (56.84)	
	No	2073 (51.66)	1940 (48.34)	

* BMI, Body mass index: Low <18.5; Normal 18.5–24.9; Overweight 25–29.9, Obese ≥30. NHANES, National Health and Nutrition Examination Survey.

**Table 3:** Association between prediabetes and work/recreational physical activity in U.S. adults from the NHANES 2015–2016.

Variables		Prediabetes		*p*
		YES	NO	
		*n* (%)	*n* (%)	
		*n* = 1150	*n* = 3331	
		25.66%	74.34%	
	2015–2016			
Work Physical Activity				0.022
	Yes	466 (23.96)	1479 (76.04)	
	No	684 (26.97)	1852 (73.03)	
Recreational Physical Activity				0.001
	Yes	478 (21.01)	1797 (78.99)	
	No	672 (30.46)	1534 (69.54)	
Gender				0.979
	Males	546 (25.68)	1580 (74.32)	
	Females	604 (25.65)	1751 (74.35)	
Age				0.001
	18–64 Years	712 (20.13)	2825 (79.87)	
	>65 Years	438 (46.40)	506 (53.60)	
Ethnic Group				0.001
	Mexican	205 (27.85)	531 (72.15)	
	Hispanic	160 (26.94)	434 (73.06)	
	White	319 (20.67)	1224 (79.33)	
	Black	313 (34.47)	595 (65.53)	
	Multiracial	153 (21.86)	547 (78.14)	
Education Level				0.001
	Elementary School	318 (32.48)	661 (67.52)	
	High School	268 (27.57)	704 (72.43)	
	Technical Education	312 (23.01)	1044 (76.99)	
	University	252 (21.47)	922 (78.53)	
				
Cholesterol				0.001
	Normal	643 (23.51)	2092 (76.49)	
	Borderline	340 (28.29)	862 (71.71)	
	High	167 (30.70)	377 (69.30)	
Hypertension				0.001
	Yes	610 (42.33)	831 (57.67)	
	No	540 (17.76)	2500 (82.24)	
BMI				0.001
	Low	10 (13.33)	65 (86.67)	
	Normal	192 (15.67)	1033 (84.33)	
	Overweight	382 (25.90)	1093 (74.10)	
	Obese	566 (33.18)	1140 (66.82)	
Waist Circumference				0.001
	Men Normal	114 (15.90)	603 (84.10)	
	Men at Risk	432 (30.66)	977 (69.34)	
	Women Normal	26 (8.67)	274 (91.33)	
	Women at Risk	578 (28.13)	1477 (71.87)	
Thyroid Disease				0.001
	Yes	968 (24.12)	3045 (75.88)	
	No	182 (38.89)	286 (61.11)	

* BMI, Body mass index: Low <18.5; Normal 18.5–24.9; Overweight 25–29.9, Obese ≥30. NHANES, National Health and Nutrition Examination Survey.

## Data Availability

Publicly available datasets were analyzed in this study. This data can be found here: https://www.cdc.gov/nchs/nhanes/index.htm.
